# Draft Genome Sequence of an IMP-7-Producing *Pseudomonas aeruginosa* Bloodstream Infection Isolate from Australia

**DOI:** 10.1128/genomeA.00596-17

**Published:** 2017-07-06

**Authors:** K. L. McCarthy, A. Jennison, A. M. Wailan, D. L. Paterson

**Affiliations:** a University of Queensland, Centre for Clinical Research, Brisbane, Queensland, Australia; b Public Health Microbiology, Forensic and Scientific Services, Queensland Health, Coopers Plains, Queensland, Australia; c Wellcome Trust Sanger Institute, Hinxton, Cambridge, United Kingdom

## Abstract

IMP-7 is one of the two IMP-type carbapenemases that in *Pseudomonas aeruginosa* are not limited to a geographic area, but it has not been previously reported in the Australian setting. We report here the draft genome sequence of an Australian *P. aeruginosa* bloodstream infection isolate that contains IMP-7.

## GENOME ANNOUNCEMENT

Metallo-beta-lactamases comprise the main group of carbapenemases found in *Pseudomonas aeruginosa*. IMP and VIM types are the most common. These genes are frequently located within class 1 integrons along with other resistance determinants. The association of the integron with a plasmid or a transposon allows transfer between bacteria. IMP-1 (for “active on imipenem”) was the first mobile *P. aeruginosa*-producing metallo-beta-lactamase discovered in Japan in 1991 (1). More recently, IMP-51 has been identified in Vietnam (2). Most of the IMP types have a defined geographical distribution apart from IMP-1 and IMP-7 (3). IMP-7 has been previously reported in Canada, central and southern Europe, Japan, Malaysia, the Czech Republic, and Saudi Arabia (4–11). This type of IMP in *P. aeruginosa* has not been previously reported in the Australian setting.

All* P. aeruginosa* bloodstream infection (BSI) isolates from patients who had blood cultures collected over a 5-year period (1 January 2008 to 31 December 2012) were retrospectively identified from five laboratories that serviced seven central tertiary care hospitals in Brisbane, Australia. Blood cultures were undertaken by the routine diagnostic laboratories using the BD BACTEC (43,003 to 1) blood culture system (BD, North Ryde, Australia) with an incubation period of up to 5 days. Upon detection, each culture-positive blood sample was inoculated onto blood, chocolate, and MacConkey agars and then incubated at 35°C in either 5% CO_2_ or aerobic conditions. After overnight incubation, the *P. aeruginosa* isolates were identified with the VITEK 2 system (bioMérieux Australia Pty. Ltd., Baulkham Hills, Australia). Antimicrobial susceptibility testing was performed by a microdilution method on the VITEK 2 system. The European Committee on Antimicrobial Susceptibility Testing breakpoints or the Clinical Laboratory Standards Institute breakpoints were utilized for interpretation (12, 13). Of the isolates that were available for further study, 24 were resistant to meropenem. To further study the BSI isolates that were carbapenem-resistant, a carbaNP test was performed (14). One isolate had a positive result.

To further study this isolate, its genome was sequenced. Paired-end libraries of whole genomic DNA of the isolate were prepared following the Nextera XT library protocol and sequenced with the Illumina NextSeq500 platform (Illumina, San Diego, CA, USA). The sequence was trimmed in CLC Genomics Workbench version 7.5.1. The sequence was *de novo* assembled using SPAdes version 3.8.1. Annotation was performed by the NCBI Prokaryotic Genome Annotation Pipeline. The draft genome was 7,097,416 bp long and had 183 contigs (total number of genes: 6,982; coding sequences: 6,913; tRNAs: 62; ncRNAs: 4). Contigs of the draft genome were submitted to the Center for Genomic Epidemiology (http://www.genomicepidemiology.org) to identify the resistance genes of the isolate with the database ResFinder version 2.1 (15). An IMP-7 gene in the isolate was identified.

### Accession number(s).

The draft genome sequence is deposited at GenBank under the accession number MWWM00000000. 

## References

[1] Watanabe M, Iyobe S, Inoue M, Mitsuhashi S 1991 Transferable imipenem resistance in *Pseudomonas aeruginosa*. Antimicrob Agents Chemother 35:147–151. doi:10.1128/AAC.35.1.147. 1901695 PMC244956

[2] Tada T, Nhung PH, Miyoshi-Akiyama T, Shimada K, Phuong DM, Anh NQ, Ohmagari N, Kirikae T 2015 IMP-51, a novel IMP-type metallo-beta-lactamase with increased doripenem- and meropenem-hydrolyzing activities, in a carbapenem-resistant *Pseudomonas aeruginosa* clinical isolate. Antimicrob Agents Chemother 59:7090–7093. doi:10.1128/AAC.01611-15. 26282421 PMC4604376

[3] Cornaglia G, Giamarellou H, Rossolini GM 2011 Metallo-beta-lactamases: a last frontier for beta-lactams? Lancet Infect Dis 11:381–393. doi:10.1016/S1473-3099(11)70056-1. 21530894

[4] Gibb AP, Tribuddharat C, Moore RA, Louie TJ, Krulicki W, Livermore DM, Palepou MF, Woodford N 2002 Nosocomial outbreak of carbapenem-resistant *Pseudomonas aeruginosa* with a new *bla*_IMP_ allele, *bla*_IMP-7_. Antimicrob Agents Chemother 46:255–258. doi:10.1128/AAC.46.1.255-258.2002. 11751148 PMC126979

[5] Hammerum AM, Jakobsen L, Hansen F, Stegger M, Sørensen LA, Andersen PS, Wang M 2015 Characterisation of an IMP-7-producing ST357 *Pseudomonas aeruginosa* isolate detected in Denmark using whole genome sequencing. Int J Antimicrob Agents 45:200–201. doi:10.1016/j.ijantimicag.2014.11.002. 25511191

[6] Ho SE, Subramaniam G, Palasubramaniam S, Navaratnam P 2002 Carbapenem-resistant *Pseudomonas aeruginosa* in Malaysia producing IMP-7 beta-lactamase. Antimicrob Agents Chemother 46:3286–3287. doi:10.1128/AAC.46.10.3286-3287.2002. 12234862 PMC128803

[7] Hrabák J, Cervená D, Izdebski R, Duljasz W, Gniadkowski M, Fridrichová M, Urbásková P, Zemlicková H 2011 Regional spread of *Pseudomonas aeruginosa* ST357 producing IMP-7 metallo-beta-lactamase in central Europe. J Clin Microbiol 49:474–475. doi:10.1128/JCM.00684-10. 20980582 PMC3020450

[8] Kouda S, Kuwahara R, Ohara M, Shigeta M, Fujiwara T, Komatsuzawa H, Usui T, Sugai M 2007 First isolation of *bla*_IMP-7 _in a *Pseudomonas aeruginosa* in Japan. J Infect Chemother 13:276–277. doi:10.1007/s10156-007-0520-0. 17721694

[9] Dortet L, Flonta M, Boudehen YM, Creton E, Bernabeu S, Vogel A, Naas T 2015 Dissemination of carbapenemase-producing *Enterobacteriaceae* and *Pseudomonas aeruginosa* in Romania. Antimicrob Agents Chemother 59:7100–7103. doi:10.1128/AAC.01512-15. 26303798 PMC4604391

[10] Hrabák J, Fridrichová M, Stolbová M, Bergerová T, Zemlickova H, Urbaskova P 2009 First identification of metallo-beta-lactamase-producing *Pseudomonas aeruginosa* in the Czech Republic. Euro Surveill 14:19102. 19215712

[11] Al-Agamy MH, Jeannot K, El-Mahdy TS, Samaha HA, Shibl AM, Plésiat P, Courvalin P 2016 Diversity of molecular mechanisms conferring carbapenem resistance to *Pseudomonas aeruginosa* isolates from Saudi Arabia. Can J Infect Dis Med Microbiol 2016:4379686. doi:10.1155/2016/4379686. PMC4997076 27597874

[12] European Committee on Antimicrobial Susceptibility Testing 2013 Breakpoint tables for interpretation of MICs and zone diameters, version 3.1 http://www.eucast.org.

[13] Clinical and Laboratory Standards Institute 2012 Performance standards for antimicrobial susceptibility testing: 22nd informational supplement. Clinical and Laboratory Standards Institute, Wayne, PA.

[14] Nordmann P, Poirel L, Dortet L 2012 Rapid detection of carbapenemase-producing *Enterobacteriaceae*. Emerg Infect Dis 18:1503–1507. doi:10.3201/eid1809.120355. 22932472 PMC3437707

[15] Zankari E, Hasman H, Cosentino S, Vestergaard M, Rasmussen S, Lund O, Aarestrup FM, Larsen MV 2012 Identification of acquired antimicrobial resistance genes. J Antimicrob Chemother 67:2640–2644. doi:10.1093/jac/dks261. 22782487 PMC3468078

